# Palmistry Notes

**Published:** 1887-10-22

**Authors:** 


### Amusements for Convalescents.

### PALMISTRY NOTES.

### By a Lady.

(*Continued from p. 426, vol. ii.*)

To those who imagine that the whole gist of the art of palmistry consists in the mere delineation of the lines in the palm, I trust that what has been said in the preceding papers on the signification of certain developments of the hand generally, will prove that there are other details of interest and importance to be considered before turning to the study of the lines in the palm. From these details we have seen that much of the character and tastes of an individual are to be gleaned from the aspect and outline of the hand and fingers.

*Types.*—There now remains but to touch briefly on the various types into which all hands are divided. First on the list stands the *elementary hand*, and as this is an uninteresting type, and one which in its entirety does not exist among educated people, a few words will suffice to treat of it. It is characterised by a thick hard palm, unusually large, frequently longer than the fingers, which are short and thick, and by a thumb which is thick and inclined to turn back. Persons with these hands are possessed of small intelligence. Naturally lazy and selfish, they never work unless compelled by necessity to do so, and they are the slaves of habit and custom from sheer incapacity to act from any inner consciousness. Music is the only refining influence to which they are susceptible. Among the low-class Tartars, Sclavs, and Laplanders the true development of the type is found. In its *unmixed* form the elementary hand is not found with us; still, in a hand of the *mixed type* some of the characteristics of the elementary hand are sometimes present, and whenever this is so, there the slothfulness of the type will materially interfere with the exercise of the better instincts and higher qualities indicated by the other developments of the mixed hand.

The *spatulate hand* is distinguished by the finger-tips being broad and spade-like, and the thumb large. Activity, mental and physical, is the leading characteristic of this hand; but when the hand is *soft*, always an indication of laziness, as we have before noticed, the active proclivities of the type will merge into a desire to be surrounded by the bustle and stir of life, without entering into it, and such subjects are wretched in a life of retirement. It is necessary to the true development of the spatulate hand that the thumb should be large, and the hand have one or both joints developed; we shall then find inventive mechanical genius, scientific ability, business capacity, perseverance, self-confidence, caution, combativeness, adminstrative powers. The best soldiers, sailors, colonists, agriculturists and mechanics, are men of the spatulate type. Freedom of thought and action in religious and political matters is also a characteristic of this type.

If in a spatulate hand the fingers are smooth and the thumb small, the indications of the type, though present, will be weakened by these conditions of the hand; and with the innate desire for movement natural to the possession of a spatulate hand, there will be a constant formation of plans and projects which will never reach maturity unless the phalange of will on the thumb be strong. Love of and research after reality in art and mechanical pursuits, with brilliancy of execution in music, are also characteristics.

The *square* or *useful hand*, with its broad, firm, hollow palm, well-proportioned root to the thumb, square finger-tips, having four distinct sides, and short nails, has many characteristics in common with the spatulate hand. Though there is less ostentation and more self-control in the square-handed subject, there is the same perseverance and foresight which characterises the spatulate type—good-sense, innate love of order and regularity, with a strong sense of the fitness of things, and of that harmony which order in the every-day things of life alone produces, justice, obedience to established authority, power of concentration, vigilance, and diplomatic gifts, are all characteristics of the square-handed subject. The strong orderliness of the type inclines also to produce tact, good taste in dress, grace of manner and deportment, with the knack of saying the right thing to the right person, and a great command over the expression of the face. With the first joint of the fingers developed there will be a love of truth in all things—fidelity, justice; with both joints, aptitude for the study of botany, history, mathematics, and an exaggeration of order and method.

(*To be continued.*)

